# Evaluation of the Novaplex^TM^ Tropical Fever Virus Assay

**DOI:** 10.3390/ijms27188398

**Published:** 2026-09-21

**Authors:** Giuseppe Sberna, Francesca Colavita, Maria Beatrice Valli, Fabiano Brillo, Fabrizio Carletti, Silvia Meschi, Cosmina Mija, Claudia Minosse, Valentina Valeriani, Gabriella Rozera, Giulia Berno, Flavia Smoquina, Lavinia Fabeni, Fabrizio Maggi, Eleonora Lalle

**Affiliations:** Laboratory of Virology and Laboratories of Biosafety, National Institute for Infectious Diseases Lazzaro Spallanzani—IRCCS, 00149 Rome, Italymariabeatrice.valli@inmi.it (M.B.V.); fabrizio.carletti@inmi.it (F.C.); claudia.minosse@inmi.it (C.M.); gabriella.rozera@inmi.it (G.R.); giulia.berno@inmi.it (G.B.);

**Keywords:** multiplex PCR, DENV, WNV, CHIKV, ZIKV, Novaplex, Dengue virus, West Nile virus, Chikungunya virus, Zika virus, arboviruses

## Abstract

Arboviral infections represent a growing challenge for global public health due to vector expansion, globalization, and climate change. Dengue (DENV), Zika (ZIKV), Chikungunya (CHIKV), and West Nile virus (WNV) often co-circulate and present overlapping clinical manifestations, making rapid and accurate etiological diagnosis difficult. In this context, multiplex real-time RT-PCR assays can support clinical management and epidemiological surveillance. In this study, analytical and preliminary retrospective comparisons of the Novaplex^TM^ Tropical Fever Virus Assay, a multiplex RT-PCR system designed for the simultaneous detection of DENV, ZIKV, CHIKV, and WNV, were performed. Analytical sensitivity was assessed using serial viral-stock dilutions and Probit regression analysis to estimate the limit of detection (LOD) at a 95% probability. The LOD was 4.41 Log copies/mL for WNV2, 3.67 for DENV2, 2.57 for ZIKV, and 3.43 for CHIKV in PBS. In spiked plasma or urine, LODs ranged from 2.04 to 4.79 Log copies/mL. No cross-reactivity with Usutu virus was observed under the conditions tested. In a retrospective comparison of 34 clinical specimens with singleplex assays, agreement was the highest for WNV, substantial for CHIKV, and limited for DENV. These findings support Novaplex as a complementary tool for arboviral diagnosis and surveillance, although further clinical studies are warranted.

## 1. Introduction

Tropical infectious diseases represent a major global health challenge, particularly in regions with high vector density, limited access to diagnostics, and frequent co-circulation of multiple viral pathogens. Arboviruses such as Dengue virus (DENV), Zika virus (ZIKV), Chikungunya virus (CHIKV), and West Nile virus (WNV), together with other emerging tropical viruses, often present with overlapping clinical manifestations, making accurate etiological diagnosis difficult. These viruses have emerged as a significant global health concern due to conditions that facilitate the spread of vectors: global travel, urbanization, and climate change [1,2,3]. In fact, the arboviruses’ contribution to global mortality and morbidity and their geographic distribution have expanded significantly in recent decades. Arboviral outbreaks increased significantly in endemic tropical and subtropical areas [4,5]. The climate change reshaping vector habitats allow vectors to expand into new geographic zones, including non-endemic temperate regions, and extending the duration of transmission periods. These rapidly evolving conditions highlight the need for a comprehensive and systematic understanding of the spatial and temporal dynamics of arboviral diseases, their vectors, and the environmental factors that drive their spread [6,7,8,9,10].

Recent epidemiological data reveal a marked increase in dengue incidence across several endemic regions. By April 2024, more than 7.6 million dengue cases and over 3000 deaths had been reported globally to WHO, while 90 countries had documented active dengue transmission. In the Region of the Americas, the 2024 case count had already exceeded the previous annual record, and the 2026 PAHO report documented 1,470,237 suspected cases. Large outbreaks were also reported in Peru in 2023 and Bangladesh, highlighting the continuing and uneven burden of DENV, with concrete risk of its expansion in temperate areas, as demonstrated with the recent outbreaks in Europe [11,12,13,14,15,16]. CHIKV and ZIKV have also generated outbreaks or a risk of local transmission beyond traditional tropical settings, including autochthonous CHIKV transmission in Italy and modeled local ZIKV transmission risk in the United States [17,18,19]. In 2017, the CHIKV outbreak in the Lazio Region involved 402 probable or confirmed autochthonous cases [19] and more recently, in 2025, local CHIKV transmission was documented in four Italian Regions (Emilia-Romagna, Veneto and Toscana) with 384 cases [20]. Moreover, in 2025, Lazio experienced its first recognized autochthonous human outbreak of WNV infection: a total of 171 cases had been confirmed [21]. As of 26 August 2026, 432 confirmed human WNV infections were reported in Italy across 17 regions [22]. Considering the Lazio region, the INMI surveillance bulletin reported 63 confirmed autochthonous WNV cases, including 31 neuroinvasive cases, 28 cases of fever, and four asymptomatic blood-donor infections [23].

In this context, rapid laboratory confirmation is essential for guiding clinical management, implementing vector-control measures, and supporting epidemiological surveillance efforts that play a crucial role in detecting arboviral outbreaks and providing data needed to assess risk, anticipate transmission, and evaluate control measures [1,24]. Because DENV, CHIKV, ZIKV, and WNV may cause overlapping and non-specific symptoms, clinical presentation alone is often insufficient for etiological diagnosis; moreover, antibody-based assays may show cross-reactivity among antigenically related orthoflaviviruses, making serology alone insufficient for definitive diagnostic confirmation [24,25]. Molecular diagnosis currently represents the reference approach for case confirmation because of its high specificity and sensitivity, particularly during the acute phase of infection when viral nucleic acids are detectable in clinical specimens. In this context, multiplex RT-PCR enables rapid simultaneous detection and differentiation of these pathogens, reducing turnaround time and supporting timely patient management, outbreak recognition, and public-health interventions.

For this reason, multiplex RT-PCR assays have increased relevance thanks to rapid turnaround time, allowing the simultaneous detection of several pathogens in a single reaction and thus improving diagnostic efficiency, particularly in resource-limited or high-throughput settings. These multiplex tests include the Novaplex^TM^ Tropical Fever Virus Assay (Novaplex; Seegene Inc., Seoul, Republic of Korea), a multiplex real-time RT-PCR system designed to detect and differentiate key tropical fever viruses within a single analytical workflow. Novaplex was selected because it enables the simultaneous detection and differentiation of DENV, ZIKV, CHIKV, and WNV in a single reaction and is compatible with standard extraction and amplification workflows used in diagnostic laboratories. This design was considered relevant for the differential diagnosis of febrile illnesses with overlapping clinical manifestations. This study aims to assess the Novaplex reliability as a diagnostic tool and to determine its suitability for integration into diagnostic algorithms for tropical viral infections, providing an analytical and a preliminary retrospective comparison of the assay.

## 2. Results

The analytical sensitivity of the Novaplex assay was assessed using serial tenfold dilutions of viral stocks for WNV lineage 2 (WNV2), ZIKV, CHIKV, and DENV serotype 2 (DENV2) in PBS. Each dilution was prepared, extracted, and tested in replicates using the Novaplex system (Table 1). The results were analyzed using a Probit regression model to estimate the limit of detection (LOD), defined as the concentration corresponding to a 95% probability of a positive result.

**Figure 1:**
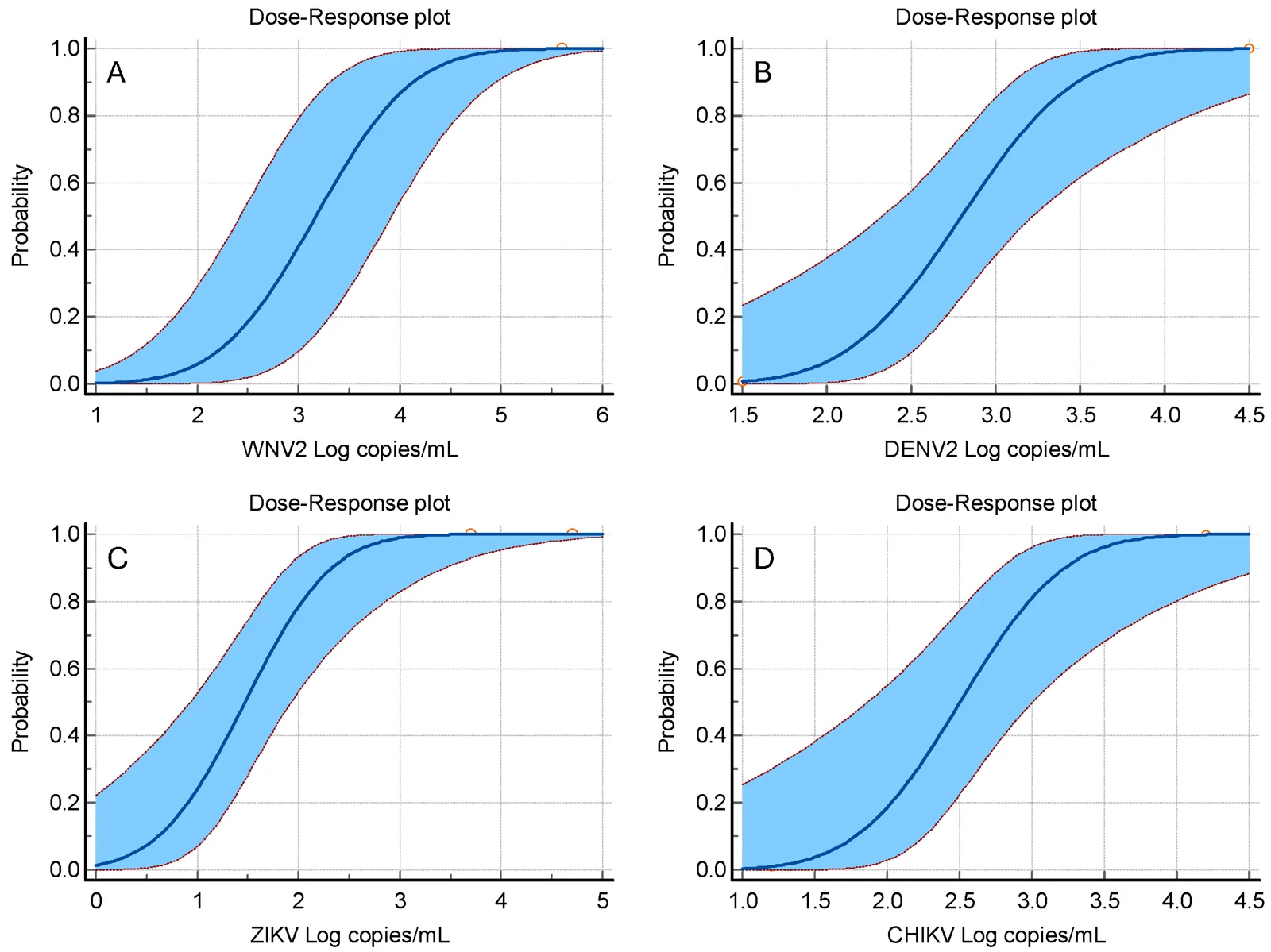
Probit analysis for Novaplex^TM^ Tropical Fever Virus Assay for WNV2 (**A**), DENV2 (**B**), ZIKV (**C**), and CHIKV (**D**) in PBS matrix.

The Probit curve generated from the WNV2 serial dilutions showed a gradual and steady decrease in the proportion of samples detected at lower concentrations. The LOD was found to be 4.41 Log copies/mL with a 95% confidence interval (95% CI): 3.55–5.26 Log copies/mL (Figure 1A). In addition, to verify the cross-reactivity of WNV2 with Usutu virus (USUV), five replicates at a concentration of 3.75 Log TCID_50_/mL were tested, yielding negative results.

Serial dilutions of DENV2 viral stock exhibited a dose–response curve that was well-fitted by the Probit model, with a linear decrease in signal intensity, yielding a LOD of 3.67 Log copies/mL (95% CI: 3.24–5.25 Log copies/mL; Figure 1B). The assay demonstrated a sensitive response to ZIKV serial dilutions, with a gradual transition between fully positive levels and dilutions near the LOD, obtained from the Probit model, that was 2.57 Log copies/mL (95% CI: 2.08–3.95 Log copies/mL; Figure 1C). For CHIKV, the dose–response curves showed a smooth trend, with a gradual loss of detection at higher dilutions. The LOD was 3.43 Log copies/mL (95% CI: 2.95–5.20 Log copies/mL; Figure 1D).

**Figure 2:**
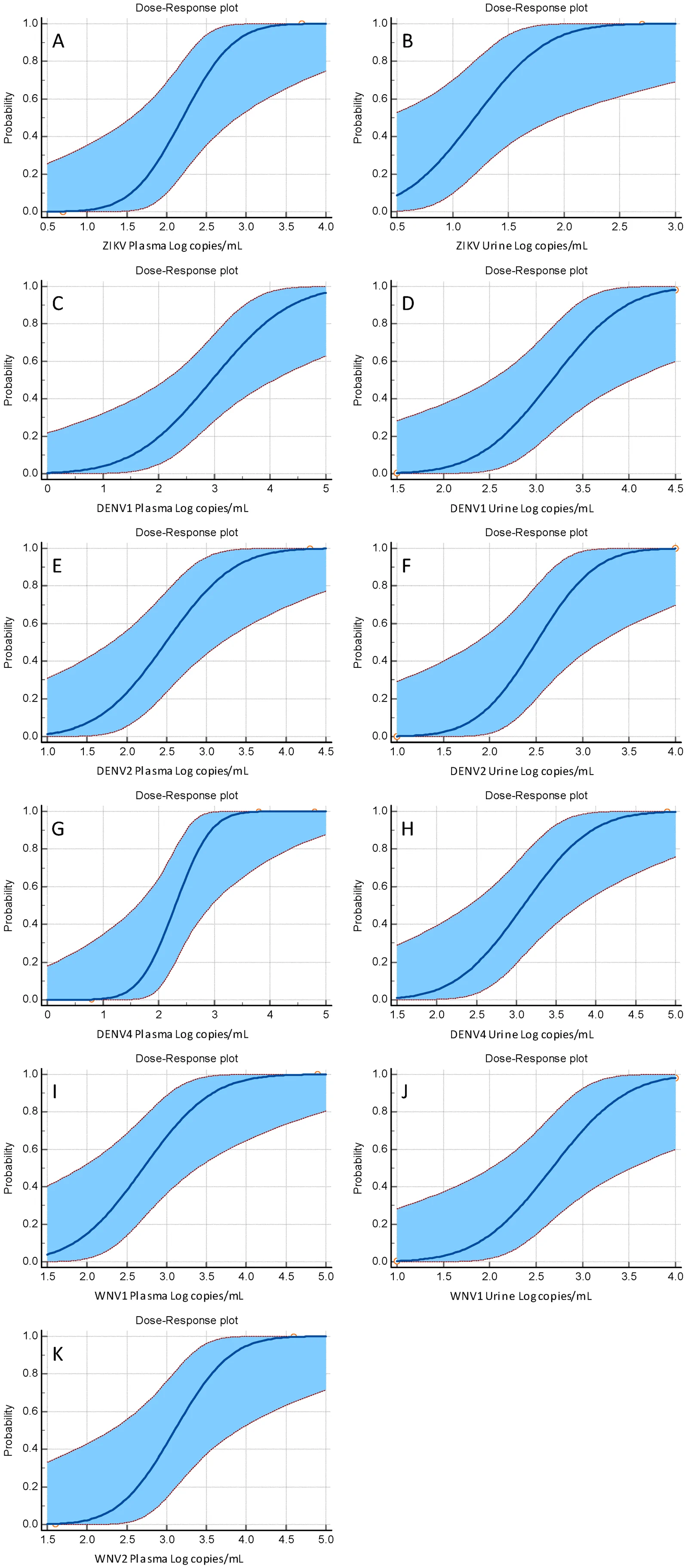
Probit analysis of the Novaplex^TM^ Tropical Fever Virus Assay for ZIKV (**A**,**B**), DENV1 (**C**,**D**), DENV2 (**E**,**F**), DENV4 (**G**,**H**), WNV1 (**I**,**J**), and WNV2 (**K**) in plasma and/or urine matrices.

Furthermore, LODs were also performed in two biological matrices routinely used for arbovirus diagnosis: plasma and urine. Tenfold serial dilutions of the viral stocks were tested (Table 2), and for each virus, Probit analysis was performed (Table 3; Figure 2).

In addition, intra-assay and inter-assay coefficients of variation (CV) were calculated for each virus/matrix combination analyzed in the study (Table 4).

Finally, a preliminary retrospective comparison of the Novaplex assay was performed using 34 biological specimens, previously tested positive for one of the following viruses: CHIKV (*n* = 12), DENV (*n* = 15), or WNV (*n* = 7). A comparison between virus-specific singleplex PCR and the Novaplex was performed (Table 5).

For CHIKV, a substantial agreement was observed between Novaplex and the RealStar^®^ Chikungunya RT-PCR Kit 2.0, with a Cohen’s kappa coefficient of 0.721 (95% CI: 0.475–0.968) (Table 5A). For DENV, a slight agreement was found between the Novaplex assay and the RealStar^®^ Dengue RT-PCR Kit 2.0 (κ = 0.124; 95% CI: 0.046–0.532; Table 5B). For WNV, an almost perfect agreement was observed between Novaplex and the RealStar^®^ WNV RT-PCR Kit 2.0 (κ = 1.000; Table 5C). Compared with the corresponding virus-specific singleplex assays, the Novaplex assay showed a positive percent agreement of 66.7% and a negative percent agreement of 100% for CHIKV. The PPV was 100%, whereas the NPV was 84.6%. For DENV, the positive percent agreement was 26.7%, while the negative percent agreement and PPV were both 100%; the NPV was 63.3%. For WNV, positive and negative percent agreement, PPV, and NPV were all 100%.

## 3. Discussion

The emergence and re-emergence of major arboviruses in tropical, subtropical, and increasingly temperate regions pose a growing challenge to global public health [4,5]. In recent decades, factors such as globalization, urbanization, and climate change have facilitated the expansion of vectors and increased viral circulation, leading to a significant rise in the incidence and geographic distribution of viruses such as DENV, ZIKV, CHIKV, and WNV [1,2,3]. In this context, multiplex RT-PCR systems represent rapid and sensitive diagnostic tools capable of distinguishing between etiological agents with overlapping clinical manifestations [5]. For these reasons, they are increasingly used to support clinical management and epidemiological surveillance, especially in endemic areas [26]. Furthermore, the main advantage of multiplex systems, namely, the ability to simultaneously analyze multiple viruses, thereby reducing time and costs, should not be overlooked.

The need for rapid and reliable discrimination among co-circulating arboviruses provided the rationale for evaluating the Novaplex^TM^ Tropical Fever Virus Assay. In PBS, the assay showed target-dependent 95% LOD ranging from 2.57 Log copies/mL for ZIKV to 4.41 Log copies/mL for WNV2. In biological matrices, LODs ranged from 2.04 to 4.20 Log copies/mL in urine and from 3.03 to 4.79 Log copies/mL in plasma. These values are broadly comparable with those reported for other multiplex arbovirus assays, although direct comparisons are limited by differences in standards, extraction procedures, sample volumes, instruments, and reporting units [24,27,28]. The variation between viral targets is consistent with the target-specific effects commonly observed in multiplex PCR systems [29]. For example, ZIKV showed a LOD of 2.57 Log copies/mL in PBS and a LOD of 2.04 and 3.03 Log copies/mL in urine and plasma, respectively. The lower ZIKV LOD in urine is consistent with studies showing that urine may provide an additional opportunity for ZIKV detection [28]. Considering DENV, the observed LOD values were 3.67 Log copies/mL in PBS, 3.33 Log copies/mL in urine, and 3.60 Log copies/mL in plasma, supporting the assay’s capability to reliably detect viral loads comparable to those detected by commercially available singleplex assays (RealStar^®^ Dengue RT-PCR Kit 2.0: 3.67 Log copies/mL [30]). The similarity of these values across the tested matrices suggests that specimen type does not significantly affect assay performance. Likewise, for WNV, comparable LOD values were obtained in PBS (4.41 Log copies/mL) and plasma (4.01 Log copies/mL). Furthermore, no cross-reactivity was observed with USUV under the tested conditions, despite the close genetic relationship between these flaviviruses. This is of great diagnostic significance particularly in European regions, where increasing seasonal USUV-WNV co-circulation has been documented [21]. The performance observed for CHIKV (LOD: 3.43 Log copies/mL) falls within the ranges reported by other multiplex methods dedicated to arboviral surveillance, which show variable sensitivity depending on viral load and biological matrix [28]. Taken together, these analytical findings also warrant attention to the Ct values observed near the LOD. In several dilution series, positive reactions at or near the lowest tested concentrations showed late Ct values, in some cases despite concentrations were not extremely low. These late-Ct results increase uncertainty in the interpretation of low-level positives and should be confirmed using a validated virus-specific assay when they are inconsistent with the clinical or epidemiological context.

A preliminary retrospective comparison was conducted using different biological specimen types that had previously tested positive for DENV, CHIKV, or WNV by routine virus-specific singleplex PCR assays. Novaplex showed slight agreement with the Altona RealStar^®^ RT-PCR Kit 2.0 for DENV detection (κ = 0.124), substantial agreement for CHIKV detection (κ = 0.721), and an almost perfect agreement for WNV detection (κ = 1.000); the complete WNV agreement is encouraging but is based on only seven positive specimens. The DENV result is the main clinical concern: Novaplex detected only 4 of 15 comparator-positive specimens, corresponding to a positive percent agreement of 26.7% (Table 5B). Although the negative percent agreement and positive predictive value were both 100%, these estimates were based on 19 comparator-negative specimens and only four Novaplex-positive specimens, respectively; the negative predictive value was only 63.3%. The low positive percent agreement and negative predictive value indicate that Novaplex did not reliably identify comparator-positive DENV specimens in this dataset and that a negative result may not exclude DENV infection. Although higher agreement has been reported for other multiplex assays, direct comparisons are limited by differences in specimen populations, extraction workflows, viral loads, and comparator methods [24,28,31]. The discrepancy may reflect low viral loads near the assay LOD, specimen matrix or storage effects, extraction efficiency, serotype distribution, limited genotype coverage, or discordance between the multiplex and comparator assays. Because the primer and probe sequences of the commercial assay were not available and genotype information was unavailable for the clinical specimens, the possibility of genotype-specific mismatches could not be evaluated.

Overall, these findings provide preliminary support for the use of Novaplex multiplex assay as a complementary tool for molecular screening and surveillance of the main arboviruses that are increasingly posing a threat also to temperate regions. Accordingly, Novaplex should not be used as a standalone test for clinical arbovirus diagnosis without confirmation by a validated virus-specific molecular assay or another appropriate confirmatory method, particularly when DENV infection is suspected or when results are late-Ct, ambiguous, discordant, or clinically inconsistent. This study supports further evaluation of Novaplex in larger prospective cohorts with balanced specimen types, quantitative viral-load data and broader representation of viral targets, and a larger number of positive clinical specimens, including all DENV serotypes and ZIKV strains.

## 4. Study Limitations

Several limitations should be acknowledged. Limited viral stocks prevented assessment of analytical sensitivity for DENV3 and CHIKV, and the WNV2 limit of detection could not be determined in urine. CSF samples were unavailable. Retrospective comparison included only 34 residual specimens, with an unbalanced distribution across viruses and matrices, no ZIKV-positive specimens, no quantitative viral-load, genotype, or serotype data available, limiting the reliable estimation of sensitivity and specificity and the generalizability of the results, and definitive conclusions regarding the assay’s clinical performance could not be drawn. The limited number of negative clinical specimens and the absence of a comprehensive exclusivity and interference assessment prevented estimation of the frequency of false-positive late-Ct results in routine use. Cross-reactivity or interference from other arboviruses, like yellow fever virus, St. Louis encephalitis virus, and Mayaro virus, and assay performance in simulated coinfections were not assessed. Finally, the lack of publicly available information on the primer and probe sequences, and genomic target regions represents an additional limitation.

## 5. Materials and Methods

### 5.1. Viruses

The following viral isolates were used to assess the analytical sensitivity of the Novaplex (Seegene Inc., Seoul, Republic of Korea): DENV1 (DENV-1/TrSt/INMI/THA/2013, EVA cat. N. 009V-03836; isolated from a clinical sample), DENV2 (New Guinea-C strain, NCPV 0006041v; provided byNCPV UK), DENV4 (DENV4/2025/INMI1; isolated from a clinical sample), CHIKV (CHIKV/ITA/lazio-INMI1-2017, EVA cat. N. 009V-02890; isolated from a clinical sample), WNV1 (Eg101; isolate provided by Public Health Agency of Canada), WNV2 (WNV2/2025/INMI1; isolated from a clinical sample), and ZIKV (ZIKV/2016/INMI1; isolated from a clinical sample).

Viral stocks were generated by culturing Vero E6 cells in Minimum Essential Medium supplemented with 10% fetal bovine serum, 1% penicillin, and 1% glutamine, and maintained at 37 °C in a 5% CO_2_ atmosphere. Cells were infected with the viral isolates within a Biosafety Level 2 (BSL-2) facility for ZIKV and USUV (used to verify the specificity and cross-reactivity of the test against WNV), while in BSL-3 for DENV1, DENV2, DENV4, CHIKV, WNV1, and WNV2. Following infection, cultures underwent three freeze–thaw cycles; cell lysates were then clarified and stored at −80 °C. Viral genome copies were quantified using QIAcuity One 5-plex Platform on the QIAcuity Nanoplate microfluidic dPCR plates (Qiagen, Hilden, Germany [32]). The reaction mixture consisted of 10 µL of 4× QIAcuity Probe PCR Kit, 0.5/0.15 µM of the viruses primer/probe mix [33,34,35,36] 10 µL of RNA, and RNase-free water, reaching the final volume to 40 µL. Data were processed with QIAcuity Suite Software version 1.1.3193. The starting concentration of each dilution series was determined by the concentration and volume of the available viral stock. Therefore, the dilution series did not begin at a common concentration for all viruses. For analytical sensitivity experiments, tenfold serial dilutions were prepared separately for each virus in PBS and, where applicable, in virus-negative plasma or urine. Each dilution was processed through the complete extraction and amplification workflow, with the number of replicates reported in Table 1 and Table 2. The LOD experiments were designed to estimate target- and matrix-specific detection limits rather than to provide a strictly head-to-head comparison of analytical sensitivity across viruses.

### 5.2. Clinical Samples

A total of 34 clinical samples collected from patients presenting at INMI for arboviruses diagnosis, from September 2017 to August 2026, were used for the preliminary retrospective comparison. After laboratory diagnosis, residual specimens were fully anonymized, and only information on their positive or negative diagnostic status was retained. Samples were stored at −80 °C until the day of analysis with the Novaplex. Specimens were selected based on the availability of residual sample volume (≥250 µL) among positive and negative samples tested for routine activities.

### 5.3. Novaplex^TM^ Tropical Fever Virus Assay

The Novaplex^TM^ Tropical Fever Virus Assay is a research-use-only (RUO) multiplex real-time RT-PCR assay manufactured by Seegene Inc. (Seoul, Republic of Korea) and distributed in Italy by Arrow Diagnostics (Genoa, Italy). The assay is designed for the simultaneous detection and differentiation of DENV, CHIKV, ZIKV, and WNV in a single reaction and includes an exogenous internal control [37]. Nucleic acids were extracted using the automated Nimbus system (Seegene). Real-time PCR analysis was carried out using the CFX96 thermal cycler (Bio-Rad Laboratories, Hercules, CA, USA [38]). Nucleic acids were extracted from 200 µL of sample and eluted in 100 µL. A volume of 5 µL of extracted nucleic acid was used in each Novaplex reaction. The assay was performed according to the manufacturer’s instructions and the corresponding instructions for use. Data analysis was conducted automatically with Seegene Viewer software (version 2.0).

### 5.4. Virus-Specific Singleplex PCR Assays

The molecular diagnosis of clinical samples was performed using virus-specific singleplex PCR assays: RealStar^®^ Chikungunya RT-PCR Kit 2.0, RealStar^®^ Dengue RT-PCR Kit 2.0, and the RealStar^®^ WNV RT-PCR Kit 2.0 (Altona Diagnostics Italia S.r.l., Segrate, Italy [30,39,40]). Total nucleic acids were extracted from 600 µL of each clinical samples using the QIAsymphony^®^ instrument with QIAsymphony^®^ DSP Virus/Pathogen Midi Kit, and eluted in 70 µL, according to the manufacturer’s instructions (Qiagen [41]). RNA (10 µL) was amplified by real-time RT-PCR using Rotor-GeneQ Real-Time cycler (Qiagen [42]).

### 5.5. Statistical Analysis

Probit regression analysis was performed to evaluate the LOD at the 95% CI by using MedCalc statistical software version 23.5.0 (MedCalc Software).

Analytical precision was assessed using independent replicates. Intra-assay precision was evaluated in a single PCR run, whereas inter-assay precision was evaluated across three independent PCR runs performed on three different days. For each virus–matrix combination and concentration, CV was calculated from the Ct values. CVs were not calculated for concentrations at which the target was not detected.

For the preliminary retrospective comparison, Novaplex results were compared with those generated by the corresponding virus-specific singleplex RT-PCR assays using two-by-two contingency tables. The singleplex RT-PCR assays results were used comparator classifications and were not considered an independent reference standard. Accordingly, the analysis was interpreted as an agreement analysis rather than as a definitive assessment of diagnostic accuracy. Given the small and unbalanced clinical specimen set, positive percent agreement, negative percent agreement, positive predictive value, negative predictive value, and Cohen’s kappa were reported descriptively; sensitivity and specificity were not estimated. Agreement between methods was assessed using Cohen’s kappa coefficient (κ), reported with its 95% CI by using GraphPad QuickCalcs (GraphPad Software [43]).

## Figures and Tables

**Table 1:** Detection limit of the Novaplex^TM^ Tropical Fever Virus Assay for WNV2, ZIKV, CHIKV, and DENV2 in PBS matrix.

(**A**)	**WNV2**			(**B**)	**DENV2**		
copies/mL	N. Total Replicates	N. Positive Replicates	Mean Ct Values (min–Max)	copies/mL	N. Total Replicates	N. Positive Replicates	Mean Ct Values (min–Max)
10^5.6^	4	4	33.5 (33.3–33.8)	10^4.5^	4	4	33.6 (33.0–34.1)
10^4.6^	4	3	38.3 (37.8–38.8)	10^3.5^	10	9	37.4 (36.6–39.73)
10^3.6^	8	8	40.5 (39.6–41.8)	10^2.5^	10	3	38.9 (38.7–39.0)
10^2.6^	8	1	41.3 (41.3)	10^1.5^	5	0	N.D.
10^1.6^	8	0	N.D. *	/	/	/	/
(**C**)	**ZIKV**			(**D**)	**CHIKV**		
copies/mL	N. Total Replicates	N. Positive Replicates	Mean Ct Values (min–Max)	copies/mL	N. Total Replicates	N. Positive Replicates	Mean Ct Values (min–Max)
10^4.7^	4	4	29.1 (28.8–29.4)	10^4.2^	10	10	32.5 (32.2–33.0)
10^3.7^	4	4	32.1 (32.0–32.3)	10^3.2^	8	7	36.3 (35.9–36.8)
10^2.7^	10	9	35.6 (35.0–36.6)	10^2.2^	6	2	39.0 (38.2–39.7)
10^1.7^	10	8	37.4 (36.6–37.8)	10^1.2^	6	0	N.D.
10^0.7^	10	0	N.D.	/	/	/	/

* N.D.: Not Determinable.

**Table 2:** Detection limit of the Novaplex^TM^ Tropical Fever Virus Assay for ZIKV (**A**,**B**), DENV1 (**C**,**D**), DENV2 (**E**,**F**), DENV4 (**G**,**H**), WNV1 (**I**,**J**), and WNV2 (**K**) in plasma and/or urine matrices.

(**A**)	**ZIKV Plasma**			(**B**)	**ZIKV Urine**		
copies/mL	N. Total Replicates	N. Positive Replicates	Mean Ct Values (min–Max)	copies/mL	N. Total Replicates	N. Positive Replicates	Mean Ct Values (min–Max)
10^3.7^	3	3	34.7 (34.4–35.0)	10^2.7^	3	3	32.9 (32.5–33.1)
10^2.7^	6	5	35.9 (33.7–37.9)	10^1.7^	6	5	33.2 (29.8–37.8)
10^1.7^	6	1	33.0 (33.0)	10^0.7^	6	1	40.2 (40.2)
10^0.7^	6	0	N.D. *	\	\	\	\
(**C**)	**DENV1 Plasma**			(**D**)	**DENV1 Urine**		
copies/mL	N. Total Replicates	N. Positive Replicates	Mean Ct Values (min–Max)	copies/mL	N. Total Replicates	N. Positive Replicates	Mean Ct Values (min–Max)
10^4.6^	3	3	31.0 (30.1–31.9)	10^4.5^	3	3	35.3 (34.7–35.7)
10^3.6^	6	4	34.7 (33.3–38.3)	10^3.5^	6	4	38.5 (30.1–38.7)
10^2.6^	6	2	37.5 (35.5–39.4)	10^2.5^	6	1	39.5 (39.5)
10^0.6^	6	1	37.9 (37.9)	10^1.5^	6	0	N.D.
10^1.6^	3	0	N.D.	\	\	\	\
(**E**)	**DENV2 Plasma**			(**F**)	**DENV2 Urine**		
copies/mL	N. Total Replicates	N. Positive Replicates	Mean Ct Values (min–Max)	copies/mL	N. Total Replicates	N. Positive Replicates	Mean Ct Values (min–Max)
10^4.3^	3	3	31.1 (30.3–31.6)	10^4.0^	3	3	34.3 (33.7–34.9)
10^3.3^	6	5	36.1 (34.6–37.7)	10^3.0^	6	5	37.2 (36.6–37.7)
10^2.3^	6	3	38.9 (38.3–39.2)	10^2.0^	6	1	39.6 (39.6)
10^1.3^	6	0	N.D.	10^1.0^	6	0	N.D.
(**G**)	**DENV4 Plasma**			(**H**)	**DENV4 Urine**		
copies/mL	N. Total Replicates	N. Positive Replicates	Mean Ct Values (min–Max)	copies/mL	N. Total Replicates	N. Positive Replicates	Mean Ct Values (min–Max)
10^4.8^	3	3	30.3 (29.8–31.1)	10^4.9^	3	3	34.3 (33.5–35.0)
10^3.8^	6	6	33.8 (33.1–34.1)	10^3.9^	6	5	31.4 (28.0–35.8)
10^2.8^	6	5	37.5 (36.1–38.4)	10^2.9^	6	3	32.1 (31.8–32.4)
10^1.8^	6	1	39.7 (39.7)	10^1.9^	6	0	N.D.
10^0.8^	3	0	N.D.	\	\	\	\
(**I**)	**WNV1 Plasma**			(**J**)	**WNV1 Urine**		
copies/mL	N. Total Replicates	N. Positive Replicates	Mean Ct Values (min–Max)	copies/mL	N. Total Replicates	N. Positive Replicates	Mean Ct Values (min–Max)
10^4.9^	3	3	31.6 (31.3–31.7)	10^4.0^	3	3	34.8 (34.6 –34.9)
10^3.9^	6	6	34.8 (34.3–35.2)	10^3.0^	6	4	35.4 (34.6–37.5)
10^2.9^	6	3	38.6 (37.1–39.6)	10^2.0^	6	1	39.0 (39.0)
10^1.9^	6	1	40.1 (40.1)	10^1.0^	6	0	N.D.
(**K**)		**WNV2 Plasma**					
copies/mL		N. Total Replicates		N. Positive Replicates		Mean Ct Values (min–Max)	
10^4.6^		5		5		31.5 (30.6–32.6)	
10^3.6^		5		4		34.9 (34.4–35.5)	
10^2.6^		5		1		38.9 (38.9)	
10^1.6^		5		0		N.D.	

* N.D.: Not Determinable.

**Table 3:** Limit of detection of the Novaplex^TM^ Tropical Fever Virus Assay for ZIKV, DENV1, DENV2, DENV4, WNV1, and WNV2 in urine and/or plasma matrices.

Virus	Matrix	LOD (Log Copies/mL)	95% CI (Log Copies/mL)	
ZIKV	Urine	2.04	1.53	6.26
	Plasma	3.03	2.53	6.02
DENV1	Urine	4.20	3.59	7.87
	Plasma	4.79	3.82	9.60
DENV2	Urine	3.33	2.83	6.32
	Plasma	3.60	2.99	6.42
DENV4	Urine	4.20	3.59	7.02
	Plasma	3.13	2.64	6.03
WNV1	Urine	3.70	3.09	7.37
	Plasma	3.83	3.22	6.87
WNV2	Plasma	4.01	3.45	7.68

**Table 4:** Intra-assay and inter-assay coefficients of variation (CV) of the Novaplex^TM^ Tropical Fever Virus Assay for WNV1, WNV2, ZIKV, DENV1, DENV2, DENV4 in urine and/or plasma matrices, and for WNV2, ZIKV, CHIKV, and DENV2 in PBS matrix.

Virus	Matrix	Copies/mL	Intra-Assay CV (%)	Inter-Assay CV (%)	Virus	Matrix	Copies/mL	Intra-Assay CV (%)	Inter-Assay CV (%)
ZIKV	PBS	10^4.7^	0.83	0.57	DENV1	PBS	/	/	/
		10^3.7^	0.13	0.50					
		10^2.7^	0.72	1.23					
		10^1.7^	1.25	1.48					
		10^0.7^	N.D. *	N.D.					
		/	/	/		Plasma	10^4.6^	3.05	/
	Plasma	10^3.7^	0.82	/			10^3.6^	0.75	N.D.
		10^2.7^	1.50	17.37			10^2.6^	7.39	N.D.
		10^1.7^	N.D.	16.88			10^0.6^	N.D.	N.D.
		10^0.7^	N.D.	N.D.			10^1.6^	N.D.	/
	Urine	10^2.7^	0.96	/		Urine	10^4.5^	1.60	/
		10^1.7^	0.47	1.63			10^3.5^	9.46	9.29
		10^0.7^	N.D.	N.D.			10^2.5^	7.39	N.D.
		/	/	/			10^1.5^	N.D.	N.D.
DENV2	PBS	10^4.5^	0.44	1.74	DENV4	PBS	/	/	/
		10^3.5^	3.86	3.00					
		10^2.5^	0.58	0.33					
		10^1.5^	N.D.	N.D.		Plasma	10^4.8^	2.21	/
	Plasma	10^4.3^	2.27	/			10^3.8^	0.67	1.87
		10^3.3^	1.69	0.21			10^2.8^	1.59	3.09
		10^2.3^	N.D.	1.22			10^1.8^	N.D.	N.D.
		10^1.3^	N.D.	N.D.			10^0.8^	N.D.	/
	Urine	10^4.0^	1.73	/		Urine	10^4.9^	2.23	/
		10^3.0^	1.06	10.50			10^3.9^	13.89	1.95
		10^2.0^	7.23	N.D.			10^2.9^	N.D.	0.95
		10^1.0^	N.D.	N.D.			10^1.9^	N.D.	N.D.
WNV1	PBS	/	/	/	WNV2	PBS	10^5.5^	0.36	0.75
							10^4.5^	1.75	N.D.
							10^3.5^	1.67	2.01
							10^2.5^	N.D.	N.D.
							10^1.5^	N.D.	N.D.
	Plasma	10^4.9^	0.66	/		Plasma	10^4.6^	3.07	2.62
		10^3.9^	0.70	1.00			10^3.6^	14.86	0.90
		10^2.9^	7.86	10.66			10^2.6^	N.D.	10.19
		10^1.9^	6.57	N.D.			10^1.6^	N.D.	N.D.
	Urine	10^4.0^	0.47	/	CHIKV	PBS	10^4.2^	0.42	0.48
		10^3.0^	N.D.	0.61			10^3.2^	0.97	0.38
		10^2.0^	N.D.	N.D.			10^2.2^	2.78	N.D.
		10^1.0^	N.D.	N.D.			10^1.2^	N.D.	N.D.

* N.D.: Not Determinable.

**Table 5:** Comparison between virus-specific singleplex PCR and the Novaplex^TM^ Tropical Fever Virus Assay. (**A**) Comparison between RealStar^®^ Chikungunya RT-PCR Kit 2.0 and Novaplex. (**B**) Comparison between RealStar^®^ Dengue Type RT-PCR Kit 2.0 and Novaplex. (**C**) Comparison between RealStar^®^ WNV RT-PCR Kit 2.0 and Novaplex. Target-negative refers to a specimen negative for the specific pathogen under comparison according to the corresponding singleplex assay; it does not imply negativity for all arboviruses included in the panel.

(**A**)		**Novaplex**		
		**Positive**	**Negative**	**Total**
RealStar^®^ Chikungunya RT-PCR Kit 2.0	Positive	8	4	12
	Negative	0	22	22
	Total	8	26	34
(**B**)		Novaplex		
		Positive	Negative	Total
RealStar^®^ Dengue RT-PCR Kit 2.0	Positive	4	11	15
	Negative	0	19	19
	Total	4	30	34
(**C**)		Novaplex		
		Positive	Negative	Total
RealStar^®^ WNV RT-PCR Kit 2.0	Positive	7	0	7
	Negative	0	27	27
	Total	7	27	34

## Data Availability

The original contributions presented in the study are included in the article; further inquiries can be directed to the corresponding author.
